# Evaluation of Dosimetric Effect of Bone Scatter on Nanoparticle-Enhanced Orthovoltage Radiotherapy: A Monte Carlo Phantom Study

**DOI:** 10.3390/nano12172991

**Published:** 2022-08-29

**Authors:** Afia Sadiq, James C. L. Chow

**Affiliations:** 1 Department of Medical Physics, Toronto Metropolitan University, Toronto, ON M5B 2K3, Canada; 2 Radiation Medicine Program, Princess Margaret Cancer Centre, University Health Network, Toronto, ON M5G 1X6, Canada; 3 Department of Radiation Oncology, University of Toronto, Toronto, ON M5T 1P5, Canada

**Keywords:** nanoparticle, nanoparticle-enhancement orthovoltage radiotherapy, skin radiotherapy, bone scatter, Monte Carlo simulation, dose enhancement, dose enhancement ratio

## Abstract

In nanoparticle (NP)-enhanced orthovoltage radiotherapy, bone scatter affected dose enhancement at the skin lesion in areas such as the forehead, chest wall, and knee. Since each of these treatment sites have a bone, such as the frontal bone, rib, or patella, underneath the skin lesion and this bone is not considered in dose delivery calculations, uncertainty arises in the evaluation of dose enhancement with the addition of NPs in radiotherapy. To investigate the impact of neglecting the effect of bone scatter, Monte Carlo simulations based on heterogeneous phantoms were carried out to determine and compare the dose enhancement ratio (DER), when a bone was and was not present underneath the skin lesion. For skin lesions with added NPs, Monte Carlo simulations were used to calculate the DER values using different elemental NPs (gold, platinum, silver, iodine, as well as iron oxide), in varying NP concentrations (3–40 mg/mL), at two different photon beam energies (105 and 220 kVp). It was found that DER values at the skin lesion increased with the presence of bone when there was a higher atomic number of NPs, a higher NP concentration, and a lower photon beam energy. When comparing DER values with and without bone, using the same NP elements, NP concentration, and beam energy, differences were found in the range 0.04–3.55%, and a higher difference was found when the NP concentration increased. By considering the uncertainty in the DER calculation, the effect of bone scatter became significant to the dose enhancement (>2%) when the NP concentration was higher than 18 mg/mL. This resulted in an underestimation of dose enhancement at the skin lesion, when the bone underneath the tumour was neglected during orthovoltage radiotherapy.

## 1. Introduction

The killing of cancer cells can effectively be improved with addition of heavy-atom nanoparticles (NPs), such as gold NPs. Studies have shown that these NPs not only increase the radiation dose delivered to the target/tumour, but also increase imaging contrast [1,2]. With enhanced image contrast the target can be better visualized by the radiation oncologist during target contouring. This results in more precise and accurate treatment planning in radiotherapy [3,4]. The reason for the dose enhancement is that the addition of the NPs makes the tumour more radiosensitive, meaning the compositional atomic number (*Z*) of the tumour cell is increased with the NP uptake [5]. As a result, when the tumour is irradiated, there is an enhancement of photoelectric effect. The enhanced yield of photoelectric electrons increases the energy deposition in the tumour cell, which in turn gives higher cancer control or killing effect [6]. 

The first study to use a high-*Z* radiosensitizer was carried out by Regulla et al. [7], who placed thin gold foil (150 μm) at depth in a polymethylmethacrylate slab, to increase the dose applied to mouse embryo fibroblasts. It was found that the cell kill rate increased by 45% in the presence of the gold foil. Furthermore, in a preclinical study, gold NPs of 1 nm diameter were applied to mice injected with subcutaneous EMT-6 mammary carcinomas. Radiotherapy using the 250 kVp photon beam was delivered to the tumours. The one-year survival curve before the addition of the gold NPs was 20%, but with the gold NPs it was 86% [8]. The gold was non-toxic and was cleared from the mice through the kidneys. Based on this study, researchers have developed a good understanding of the radiosensitization properties of high-*Z* NPs in dose enhancement [9,10]. In the above study, mammographic images were taken to observe the uptake of the NPs in the cells. From these images, it was shown that the rate of NP uptake was faster in cancerous cells than in healthy cells [11]. The cellular uptake of NP occurred mainly through energy-dependent receptor-mediated endocytosis. This resulted in the NPs accumulating in the endosome–lysosomes [12]. Following this preliminary study, Hainfeld et al. performed a preclinical experiment on the addition of gold NPs for radio-resistant brain squamous carcinoma [13]. It was found that by varying dose fractionation, radiation beam energy, and radiation dosimetry, adding gold NPs could increase the treatment outcome of aggressive squamous cell carcinoma. In a follow-up study to Hainfeld et al. [8], Monte Carlo simulations were performed to further quantify the dose enhancement properties of gold NPs. With the help of Monte Carlo codes (e.g., BEAM and DOSXYZ combined with EGS4), the dose enhancement ratio (DER) due to the addition of gold NPs was quantified based on several parameters; photon beam energies of 140 kVp, 4 MV, and 6 MV spectra with and without flattening filters, and gold NP concentrations of 7, 18, and 30 mg/mL [14]. These studies inspired many researchers to investigate the role of NPs in radiotherapy, using different types of radiation beams and delivery methods [15,16,17,18]. This has included skin therapy using orthovoltage photon beams [19]. 

According to the World Health Organization, the incidence of skin cancers has been increasing over the past few decades, with around 2–3 million cases occurring annually around the world [20]. With early detection and treatment, skin cancer is highly treatable, with radiotherapy being the favoured option. Radiotherapy can have a 90% success rate on skin cancers, with few to no side effects. The side effects are usually mild and last for a few weeks, until healthy cells grow back [21]. Generally, skin cancer is treated using orthovoltage photon beams. Orthovoltage radiotherapy refers to treatment using photon beam energies in the 100–300 kVp range. These kV photon beams are produced by an orthovoltage x-ray unit with a treatment cone and cutout, to conform the beams to the target of skin lesion. The radiation dose at the target skin surface is prescribed by the radiation oncologist. The required monitoring unit or treatment time is calculated by the radiotherapists and medical physicists using pre-measured dose data such as backscatter factor, relative exposure factor, and percentage depth dose [22]. Such calculations can be carried out manually using dose data tables or automatically using computer software [23].

In this study, we focused on the effect of bone scatter on dose enhancement, when NPs were added to skin lesions in NP-enhanced orthovoltage radiotherapy. Since the monitoring unit or treatment time calculation neglects the bone underneath the skin lesion in certain sites including the forehead, cheek, chest wall, and knee, the effect of bone scatter is neglected in the prescription dosimetry. When a skin lesion in the above sites is irradiated by a kV photon beam, a loss of backscatter occurs as the bone’s inhomogeneity reduces the surface dose in the treatment. In current treatment modalities, dose reduction is not considered in the dose calculation. Thus, there may be an overestimation of the prescribed dose. Butson et al. [24] measured dose reduction caused by bone inhomogeneity, using a Gulmay D3300 X-ray machine (Gulmay Medical, Surrey, UK). In that study, an Attix parallel plate ionization chamber and EBT GAFCHROMIC film were used when taking measurements. Butson et al. [24] found that in some cases the presence of bone affected the backscattered dose by up to 12.5% of the surface dose (cone size diameter = 10 cm and 100 kVp beam). 

Monte Carlo simulations are suitable for predicting macroscopically and nanoscopically the radiation dosimetry in NP-enhanced radiotherapy [25]. The Monte Carlo method is a mathematical algorithm based on random sampling to predict a numerical solution when the absolute solution is very difficult to determine. Different Monte Carlo codes such as the EGSnrc and Geant4 have been used and proved to be effective in NP dosimetry [26,27]. Studies have investigated dose enhancement, image contrast enhancement, and DNA damage in NP-enhanced radiotherapy using various radiation beams such as photon, proton, or electron beams [28,29,30]. Since the accuracy of a Monte Carlo simulation depends on the number of histories, a long computing time is therefore an issue in the simulation when aiming to obtain an accurate result. With recent advances in high-performance computing, the difficulty of long computing time was solved by using new computing technologies including cell-processor, cloud, and grid computing [31]. In this current study, Monte Carlo simulations were carried out to determine the DER at the skin lesion, when the bone underneath the skin was or was not neglected during NP-enhanced orthovoltage radiotherapy. This study focuses on predicting the physical dose enhanced at the skin lesion, using Monte Carlo simulation. The biological dose that affects cancer-cell killing and clinical outcome is not within the scope of this study [32,33]. 

## 2. Materials and Methods 

### 2.1. Monte Carlo Simulation

For the Monte Carlo simulation using Electron Gamma Shower, the National Research Council (EGSnrc) code developed by the National Research Council of Canada was used to predict the radiation dose in this study [34]. The simulations were carried out using photon beams of 105 kVp (HVL: 3.2 mm Al) and 220 kVp (HVL: 1.7 mm Cu), produced by a Gulmay D3550 X-ray unit (Gulmay Medical, Surrey, UK) for gold, platinum, silver, iodine, and iron oxide NPs. The EGSnrc code is a software toolkit for performing Monte Carlo simulations of ionising radiation travelling through varying materials. It was developed in Canada, and is an improved version of the EGS package from the 1970s that was developed at the Stanford Linear Accelerator Centre [35]. As the name suggests, the program models certain “showers” that are generated as a result of electrons and photons transported in a medium. The EGSnrc code contains numerous applications that employ radiation physics to calculate specific quantities. These codes have been developed by numerous authors in order to support the large user community. The applications of the EGSnrc can be divided into two categories; Fortran codes and C++ codes (egs++). The Fortran codes consist of many applications, including the BEAMnrc and DOSXYZnrc that were used in this study [36].

Monte Carlo simulations are well-known as the benchmark for predicting radiation dosimetry in heterogeneous materials such as soft tissue, lung, bone, and metal [35]. The simulation has been used successfully to study metal and bone scatter in radiotherapy, and dose enhancement at the tumour in NP-enhanced radiotherapy involving kV and MV photon beams [37]. In this study, a Monte Carlo simulation using the macroscopic approach was carried out to predict dose enhancement at the skin lesion, when the bone underneath the tumour was or was not considered in the simulation model. In current skin-therapy practice regarding the dose prescribed at the patient’s skin, any bone underneath the lesion is neglected. Results from the simulation were used to assess the impact of bone scatter on the dose enhancement in NP-enhanced orthovoltage radiotherapy.

### 2.2. Simulation Model and Geomtry

**Figure 1:**
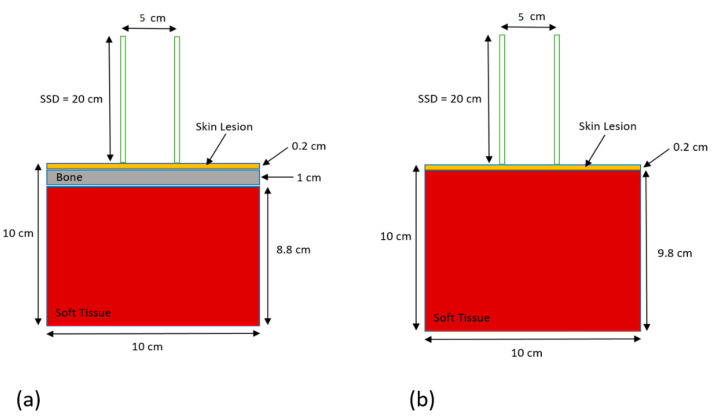
Schematic diagrams (not to scale) showing the heterogeneous phantoms used in Monte Carlo simulations, (**a**) with and (**b**) without a bone between the skin lesion and the soft tissue. The dimensions of the phantoms were equal to 10 × 10 × 10 cm^3^. The phantoms were irradiated by the 105 and 220 kVp photon beams with circular cone applicator (5 cm diameter) attached to the phantom’s surface. The source-to-surface distance (SSD) was equal to 20 cm, mimicking the clinical dose delivery.

The simulation geometry using two heterogeneous phantoms is shown in Figure 1. Figure 1a shows a phantom with a layer of soft tissue (skin lesion) on top of a bone with thickness equal to 1 cm. The thickness of the skin lesion was equal to 0.2 cm. The bone was on top of a slab of soft tissue with a thickness of 8.8 cm. The dimensions of the phantom were 10 × 10 × 10 cm^3^ and it was designed to mimic a forehead treatment site. The phantom surface (skin) was placed in contact with a circular treatment cone of 5 cm diameter with source-to-surface distance equal to 20 cm. The cone was made of copper, steel, and poly(methyl methacrylate). The 105 and 220 kVp photon beams were generated by the Gulmay D3550 X-ray unit (Gulmay Medical, Surrey, UK) to irradiate the phantom. The kV photon beams were based on the Monte Carlo phase-space files of the treatment unit with the corresponding beam energy, quality, and geometry [38]. The phase-space files were generated using the EGSnrc-based BEAMnrc code [39]. For the heterogeneities in the phantom, the ICRUBONE521ICRU from the PEGS4 cross-section data file was selected to mimic the bone, and the ICRPTISSUE521ICRU was selected to mimic the skin lesion and soft tissue [34]. These standard materials are available in the PEGS4 data library in the simulation code. To simulate the skin lesion with the addition of NPs and model the material with NPs, cross-section datasets for various NP concentrations were created using the EGSnrc-based PEGS4 code. The datasets contained physical information of particle interactions of gold, platinum, silver, iodine, and iron oxide NPs at concentrations of 3, 7, 18, 30, and 40 mg/mL [19]. This range of concentration was selected according to small-animal experiments in nanoparticle-enhanced radiotherapy [8]. It was assumed that there was no biological washout of NPs during the simulation. Radiation dose was calculated at the skin lesion layer using the DOSXYZnrc code [40], and 150 million histories were run for each simulation. Under this number of histories, the uncertainty of calculated dose in the simulation was less than 1%. The energy cut-offs for the electron and photon transport were set to 521 and 1 keV, respectively. In the simulation, the PRESTA II was used for the electron-step algorithm, and the feature options of spin effect, bound Compton scattering, Rayleigh scattering, atomic relaxation, and electron impact ionization were all employed in the simulation using the kV photon beams [34]. For this phantom (Figure 1a), various kinds of doses were added at the skin lesion layer: with and without addition of NPs, with various metallic elements, and with various concentrations and photon beam energies. To investigate the dependence of dose enhancement on the effect of bone scatter, Figure 1b mimicked the case when the bone underneath the skin lesion was neglected in radiotherapy. In Figure 1b, the bone layer in Figure 1a was replaced by soft tissue. This is the clinical situation when the monitor unit or treatment time is calculated with the dose prescribed at the patient’s skin [22]. In this event, the material underneath the skin lesion was assumed to be soft tissue or water during the treatment. The same simulations were carried out as per the phantom in Figure 1a. By comparing the dose enhancements at the skin lesions in Figure 1a,b, the difference of dose enhancement due to the presence of bone scatter was determined.

### 2.3. Dose Enhancement Ratio (DER)

The dose enhancement in the presence of NPs at the skin lesion is expressed as DER [41]:

(1) $$Does\;Enhancement\;Ration\;\left(\mathrm{DER}\right)=\frac{Does\;at\;the\;skin\;lesion\;with\;nanoparticle\;addition}{Dose\;at\;the\;skin\;lesion\;without\;nanoparticle\;addition}$$

When there was no NP added to the skin lesion in the simulation model, the material of the skin layer was set to soft tissue. This resulted in a DER value calculation of one. Because the addition of NPs can increase the dose at the skin lesion layer due to the enhancement of photoelectric effect, the dose at the skin lesion was higher than the same layer without added NPs. This caused the DER value exceed one, reflecting the dose enhancement.

## 3. Results

**Figure 2:**
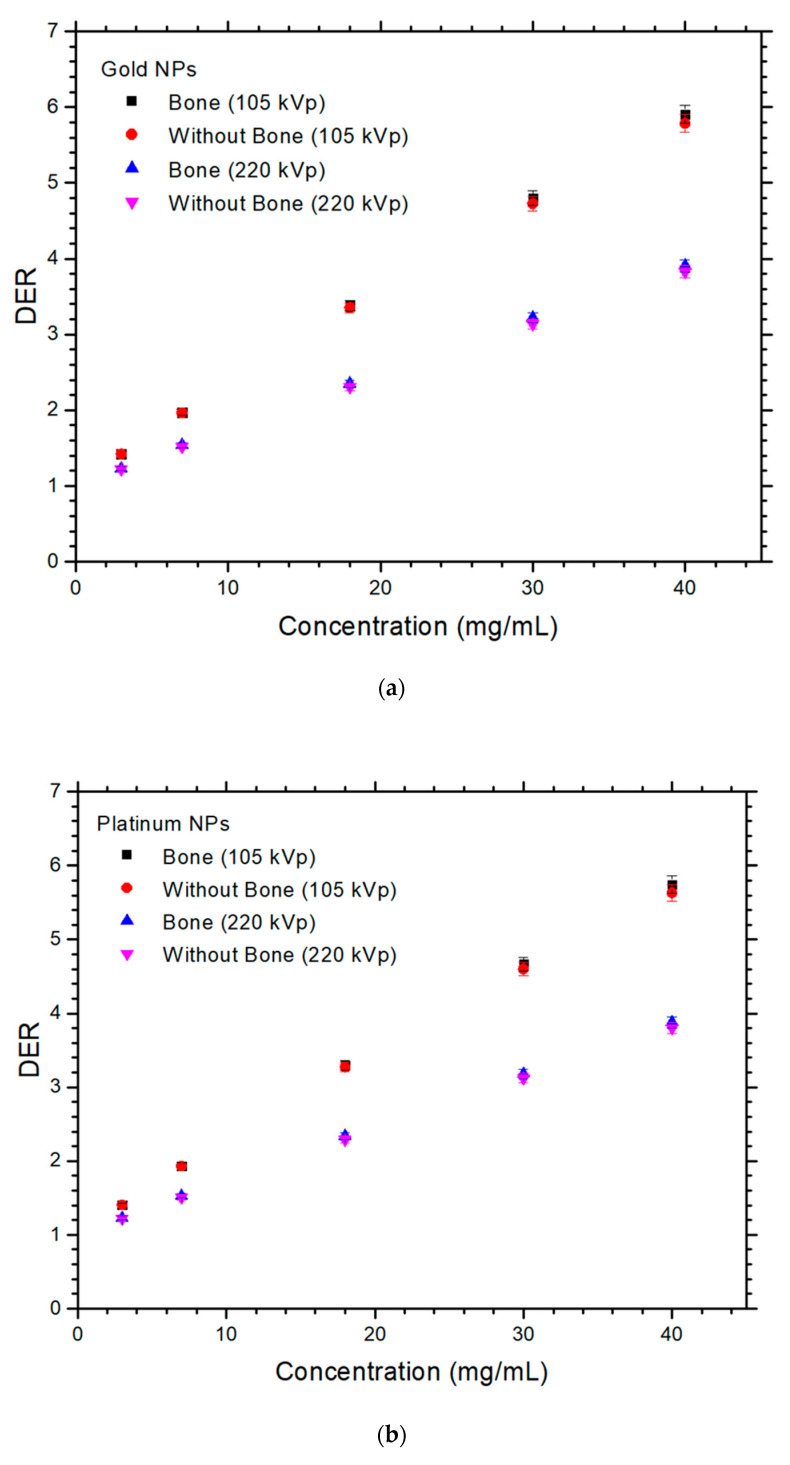

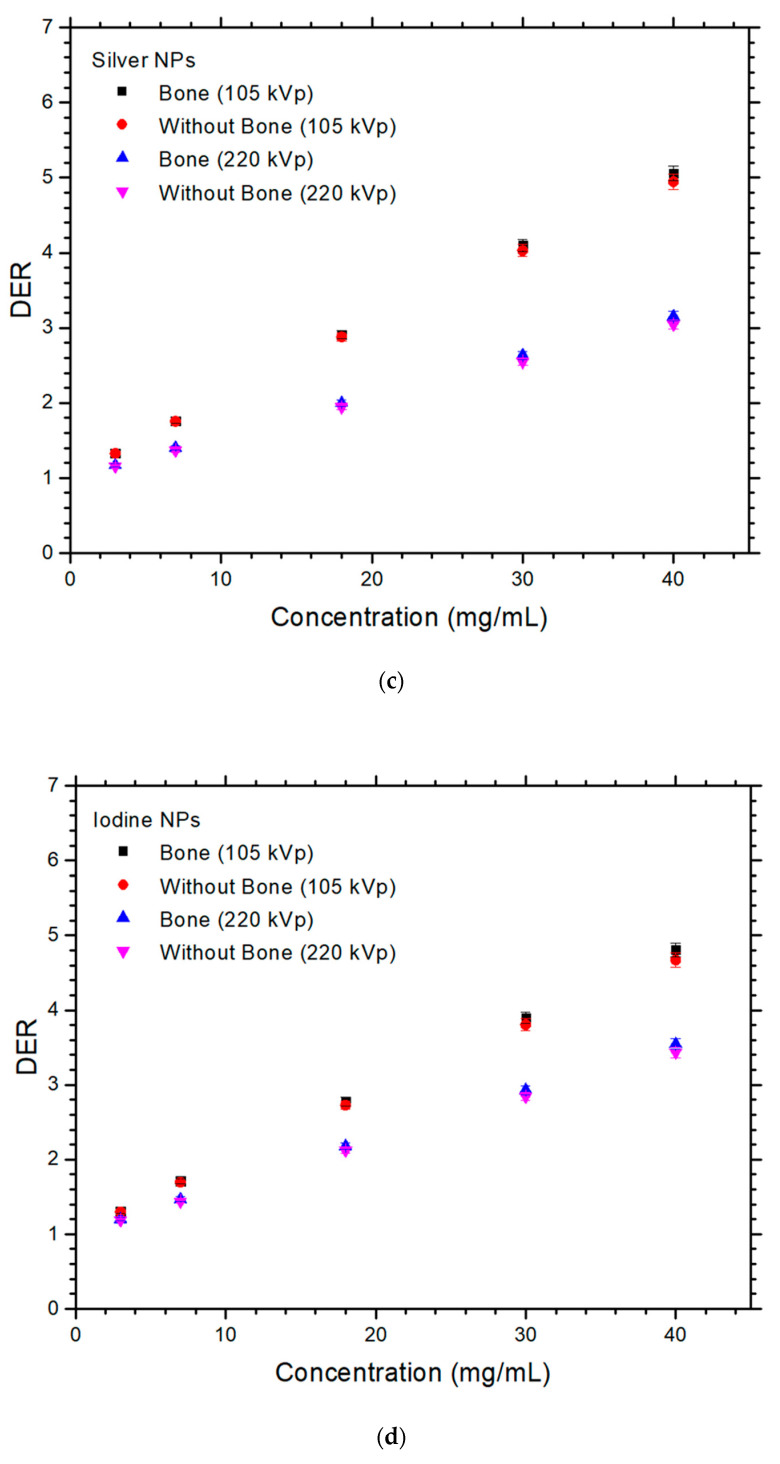

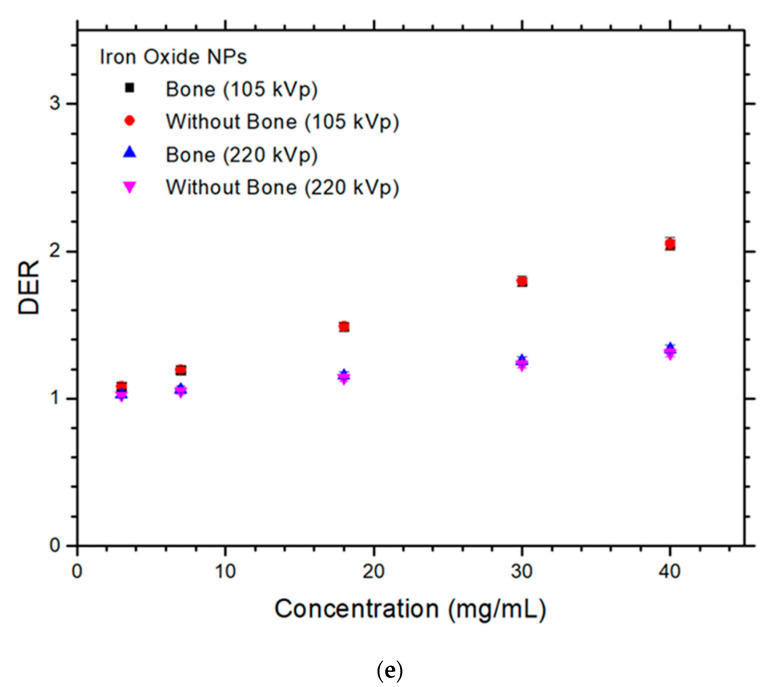
Relationships of dose enhancement ratio and NP concentration (mg/mL) with variations of photon beam energies (105 and 220 kVp) and presence of bone for (**a**) gold, (**b**) platinum, (**c**) silver, (**d**) iodine, and (**e**) iron oxide NPs in Monte Carlo simulations.

The relationships between DER and NP concentration with variable presence of bone and photon beam energy are shown in Figure 2a–e, for the gold, platinum, silver, iodine, and iron oxide NPs. The uncertainty of the calculated DER was equal to 2% based on the radiation dose determined from Monte Carlo simulation. It can be seen in Figure 2 that all DER values were higher than one, showing that the addition of NPs to the skin lesion enhanced the dose during orthovoltage radiotherapy. The maximum DER value shown in Figure 2a was 5.91, for the phantom with bone irradiated by the 105 kVp photon beam. This DER value was larger than the maximum DER values of 5.75, 5.05, 4.81, and 2.05 shown in Figure 2b–e with the same phantom geometry and beam energy. Similarly, the minimum DER values were 3.83, 3.79, 3.43, 3.04, and 1.31 for the gold, platinum, silver, iodine, and iron oxide NPs, respectively, in the phantom without bone irradiated by the 220 kVp photon beam (Figure 2). The differences of DER with and without the presence of bone, shown in Figure 2, were calculated for different NPs, NP concentrations, and photon beam energies. These results are shown in Table 1. The differences of DER for the gold, platinum, silver, iodine, and iron oxide NPs ranged from 0.04–2.08, 0.06–2.01, 0.16–2.22, 0.37–2.79, and 0.06–0.62 using the 105 kVp photon beam, and 0.77–2.98, 0.61–3.08, 1.55–3.55, 1.17–3.23, and 0.4–2.12 using the 220 kVp beam, respectively.

## 4. Discussion

### 4.1. Dependences of DER on the NP and NP Concentration

For the different elemental NPs used in this study, it is found that gold NPs performed best with maximum DER value of 5.9 using the maximum NP concentration (40 mg/mL) for the phantom with bone (Figure 1a) irradiated by the 105 kVp photon beam. This was followed by the platinum NPs with DER value equal to 5.75, then silver NPs, iodine NPs, and iron oxide NPs with DER values equal to 5.06, 4.81, and 2.05, respectively. It is seen that the DER value increased with the increase of *Z* of the NPs. This is because the dose enhancement was due to the increased energy deposition at the lesion, resulting from the increased number of secondary electrons. These electrons are generally produced by photoelectric interaction, which is dominant in the kV energy range. The increase of compositional *Z* at the skin lesion enhanced the photoelectric effect, resulting in an increase of photoelectric electron yield [42]. As the cross-section of the photoelectric effect is proportional to *Z^n^* (*n* varies between 4 and 5), it can be seen that the higher the *Z*, the higher the DER value of the lesion with the addition of NPs. Therefore, gold (*Z* = 79) had the highest DER value, followed by platinum (*Z* = 78), silver (*Z* = 53), iodine (*Z* = 47), and iron oxide (*Z* = 23).

In Figure 2, DER values of the NPs were found to increase with concentration. This is because higher concentrations of NPs contain more number of particles. These NPs generate more secondary electrons (e.g., photoelectric and Auger electrons) when interacting with the kV photons through the photoelectric and Auger effect [43]. These extra electrons deposit more energy in the lesion to enhance the dose. Moreover, such an increase of DER value is more sensitive for NPs with higher *Z* than lower. For example, considering the phantom with bone (Figure 1a) irradiated by the 105 kVp photon beam, gold NPs had their DER value increased at a rate of 0.12 (mg/mL)^−1^. This was higher than that of the iron oxide NPs, at a rate of 0.026 (mg/mL)^−1^. 

### 4.2. Dependences of DER on the Presence of Bone and Photon Beam Energy

When bone is present underneath the skin lesion in photon beam irradiation, the radiation dose is reduced as the bone decreases the backscatter to the skin layer [24,38]. This effect is more significant when the NP concentration is high (e.g., 40 mg/mL). Moreover, this effect is more significant when a kV photon beam is used. Photoelectric effect is dominant in the kV energy range, and the photoelectric interaction increases with an increase of *Z*. Therefore, more photons are absorbed by the bone than the soft tissue, because bone has a higher *Z*. This results in a lower dose at the skin lesion when the presence of bone is considered [38]. However, in the calculation of DER using the bone phantom, the effect of reduction of skin dose was cancelled out in Equation (1) because both the numerator and denominator contained bone in the phantom.

When NPs were added to the skin lesion in this study, the dose enhancement with bone was higher than that without bone, as shown in Figure 2. For example, when the NP concentration was equal to 40 mg/mL using the 220 kVp photon beam, the DER value for the gold NPs was 3.90 with bone and 3.78 without bone. Similar trends of results were found with other NPs, NP concentrations, and beam energies. When NPs were added to the skin lesion, the number of photons arriving at the bone layer was decreased because the NP–skin lesion layer acted as a shield to reduce the photon fluence to the bone [44]. This caused a higher DER value as more NPs were added to the skin lesion with bone compared with that without bone underneath the skin. Therefore, by considering dose enhancement due to the addition of NPs, and the “shielding effect” on the bone due to the skin layer with added NPs, the DER value was found to be slightly increased when the bone’s effect on dose enhancement due to the addition of gold NPs was considered in the calculation.

In Figure 2, the DER values for NPs using the 105 kVp photon beams were higher than those for 220 kVp. In addition, the higher the NP concentration, the larger was the difference between the DER values at the two beam energies. As the cross-section of the photoelectric effect is inversely proportional to the energy of the incident photon, the lower energy of 105 kVp resulted in a larger cross-section and hence a higher photoelectric electron yield compared with 220 kVp. This was reflected in the DER values of the corresponding photon beam energies in Figure 2.

### 4.3. Difference of DER with and without Presence of Bone

The effect of bone scatter on the variation in DER values is shown in Table 1, for different NPs, NP concentrations, and photon beam energies. The differences of DER were in the range 0.04% to 3.55%, and it was found that the value was lower for lower NP concentrations (e.g., 0.04–1.55% at 3 mg/mL), and higher for higher NP concentrations (e.g., 2.01–3.55% at 40 mg/mL). Since the uncertainty in the DER calculation was 2%, most differences of DER values (with or without bone scatter) were within the simulation uncertainty, except when the values were higher than 2%, which mostly occurred at higher NP concentrations (>18 mg/mL). In this event, the effect of bone scatter became significant enough to affect the dose enhancement at the skin lesion in radiotherapy. It was found that underestimation of the DER value for skin when neglecting the bone scatter would be more significant at higher NP concentrations.

## 5. Conclusions

Present trends in orthovoltage radiotherapy include enhancement of the effect of the therapy by adding NPs to the skin lesion. However, in absence of NPs the presence of bone tissue under the skin decreases the effective dose in the skin lesion. The present study addresses the question whether the presence of bone under the skin would increase or decrease the effective dose in the presence of NPs in the skin. By using Monte Carlo simulation methods with phantom models, it was found that in the presence of NPs the effective dose in the presence of bone was increased compared to the case without bone. The effect was better measurable for NP concentrations above 18 mg/mL. This threshold depends on the *Z* of the NP material: *Z* (26–79), and threshold (2.12–3.55%). This result should be taken into account in future clinical applications. It should be noted that the DER value for the bone phantom was independent of the reduction of skin dose due to the presence of bone, but the bone effect on the dose enhancement was due to the addition of gold NPs. Higher DER values were found when the bone scatter was considered in the simulation. Moreover, the DER values increased with higher *Z* of NPs, higher NP concentration, and lower photon beam energy. It is concluded that in NP-enhanced orthovoltage radiotherapy, dose enhancement may be slightly underestimated when bone scatter is neglected, particularly when the skin lesion has a high NP concentration. Considering dose enhancement with and without bone, the DER differences were found to vary between 0.04% and 3.55% for different NPs, NP concentrations, and beam energies. DER difference > 2% should be considered as unneglectable in clinical practice. 

One limitation of this work is that variation of DER by NP size was not considered in this study. This is because it remains too complicated to create a realistic Monte Carlo model with billions of NPs in the simulation volume. Future work will include clinical results from real treatment of orthovoltage radiotherapy on real patients, where the material of the voxels inside the bone structure will be replaced by tissue material. The computed tomography image set used can verify the results from the Monte Carlo test, for effective dosimetry in orthovoltage radiotherapy.

## Figures and Tables

**Table 1:** Difference of DER (%) between bone under the skin and no bone under the skin in the Monte Carlo simulation, using various NPs, NP concentrations, and photon beam energies. Differences of DER values are marked in bold where statistically significant (*p* < 0.05).

Photon Beam Energy	105 kVp					220 kVp				
Concentration (mg/mL)	Gold NP	Platinum NP	Silver NP	Iodine NP	Iron Oxide NP	Gold NP	Platinum NP	Silver NP	Iodine NP	Iron Oxide NP
3	0.04	0.06	0.16	0.37	0.06	0.77	0.61	1.55	1.17	0.40
7	0.18	0.14	0.43	0.93	0.14	1.27	1.38	**2.32**	1.87	0.95
18	0.75	0.71	1.06	1.74	0.29	1.94	**2.07**	**2.77**	**2.51**	1.49
30	1.49	1.46	1.67	**2.33**	0.47	**2.61**	**2.86**	**3.29**	**2.98**	1.93
40	**2.08**	**2.01**	**2.22**	**2.79**	0.62	**2.98**	**3.08**	**3.55**	**3.23**	**2.12**

## References

[1] Siddique S., Chow J.C.L. (2020). Application of Nanomaterials in Biomedical Imaging and Cancer Therapy. Nanomaterials.

[2] Chithrani D.B., Jelveh S., Jalali F., Van Prooijen M., Allen C., Bristow R.G., Hill R.P., Jaffray D.A. (2010). Gold nanoparticles as radiation sensitizers in cancer therapy. Radiat. Res..

[3] Abdulle A., Chow J.C.L. (2019). Contrast enhancement for portal imaging in nanoparticle-enhanced radiotherapy: A Monte Carlo phantom evaluation using flattening-filter-free photon beams. Nanomaterials.

[4] Albayedh F., Chow J.C.L. (2018). Monte Carlo simulation on the imaging contrast enhancement in nanoparticle-enhanced radiotherapy. J. Med. Phys..

[5] Chow J.C.L., Torres Martinez L.M., Vasilievna Kharissova O., Ildusovich Kharisov B. (2017). Application of Nanoparticle Materials in Radiation Therapy. Handbook of Ecomaterials.

[6] Retif P., Pinel S., Toussaint M., Frochot C., Chouikrat R., Bastogne T., Barberi-Heyob M. (2015). Nanoparticles for radiation therapy enhancement: The key parameters. Theranostics.

[7] Regulla D.F., Hieber L.B., Seidenbusch M. (1998). Physical and biological interface dose effects in tissue due to X-ray-induced release of secondary radiation from metallic gold surfaces. Radiat. Res..

[8] Hainfeld J.F., Slatkin D.N., Smilowitz H.M. (2004). The use of gold nanoparticles to enhance radiotherapy in mice. Phys. Med. Biol..

[9] Moore J., Chow J.C.L. (2021). Recent progress and applications of gold nanotechnology in medical biophysics using artificial intelligence and mathematical modeling. Nano Express.

[10] Siddique S., Chow J.C.L. (2020). Gold nanoparticles for drug delivery and cancer therapy. Appl. Sci..

[11] Karathanasis E., Suryanarayanan S., Balusu S.R., McNeeley K., Sechopoulos I., Karellas A., Annapragada A.V., Bellamkonda R.V. (2009). Imaging nanoprobe for prediction of outcome of nanoparticle chemotherapy by using mammography. Radiology.

[12] Oh N., Park J.H. (2014). Endocytosis and exocytosis of nanoparticles in mammalian cells. Int. J. Nanomed..

[13] Hainfeld J.F., Dilmanian F.A., Zhong Z., Slatkin D.N., Kalef-Ezra J.A., Smilowitz H.M. (2010). Gold nanoparticles enhance the radiation therapy of a murine squamous cell carcinoma. Phys. Med. Biol..

[14] Cho S.H. (2005). Estimation of tumour dose enhancement due to gold nanoparticles during typical radiation treatments: A preliminary Monte Carlo study. Phys. Med. Biol..

[15] Huynh N.H., Chow J.C.L. (2021). DNA dosimetry with gold nanoparticle irradiated by proton beams: A Monte Carlo study on dose enhancement. Appl. Sci..

[16] Jabeen M., Chow J.C.L. (2021). Gold Nanoparticle DNA Damage by Photon Beam in a Magnetic Field: A Monte Carlo Study. Nanomaterials.

[17] Haume K., Rosa S., Grellet S., Śmiałek M.A., Butterworth K.T., Solov’yov A.V., Prise K.M., Golding J., Mason N.J. (2016). Gold nanoparticles for cancer radiotherapy: A review. Cancer Nanotechnol..

[18] Peukert D., Kempson I., Douglass M., Bezak E. (2018). Metallic nanoparticle radiosensitisation of ion radiotherapy: A review. Phys. Med..

[19] Zheng X.J., Chow J.C.L. (2017). Radiation dose enhancement in skin therapy with nanoparticle addition: A Monte Carlo study on kilovoltage photon and megavoltage electron beams. World J. Radiol..

[20] Radiation: Ultraviolet (UV) Radiation and Skin Cancer. https://www.who.int/news-room/questions-and-answers/item/radiation-ultraviolet-(uv)-radiation-and-skin-cancer.

[21] Fischbach A.J., Sause W.T., Plenk H.P. (1980). Radiation therapy for skin cancer. West. J. Med..

[22] Chen Z., Chow J.C.L., Sun A., Nagar H., Stevens K.R., Knisely J.P.S., Khan F.M., Sperduto P.W., Gibbons J.P. (2022). Cancers of the Skin, Including Mycosis Fungoides. Khan’s Treatment Planning in Radiation Oncology.

[23] Pearse J., Chow J.C.L. (2020). An Internet of Things App for Monitor Unit Calculation in Superficial and Orthovoltage Skin Therapy. IOP SciNotes.

[24] Butson M.J., Cheung T., Peter K.N. (2008). Measurement of dose reductions for superficial x-rays backscattered from bone interfaces. Phys. Med. Biol..

[25] Chow J.C.L. (2018). Recent progress in Monte Carlo simulation on gold nanoparticle radiosensitization. AIMS Biophys..

[26] Sheeraz Z., Chow J.C.L. (2021). Evaluation of dose enhancement with gold nanoparticles in kilovoltage radiotherapy using the new EGS geometry library in Monte Carlo simulation. AIMS Biophys..

[27] Sakata D., Kyriakou I., Tran H.N., Bordage M.C., Rosenfeld A., Ivanchenko V., Incerti S., Emfietzoglou D., Guatelli S. (2019). Electron track structure simulations in a gold nanoparticle using Geant4-DNA. Phys. Med..

[28] Chun H., Chow J.C.L. (2016). Gold nanoparticle DNA damage in radiotherapy: A Monte Carlo study. AIMS Bioeng..

[29] Sharma M., Chow J.C.L. (2020). Skin dose enhancement from the application of skin-care creams using FF and FFF photon beams in radiotherapy: A Monte Carlo phantom evaluation. AIMS Bioeng..

[30] Sotiropoulos M., Henthorn N.T., Warmenhoven J.W., Mackay R.I., Kirkby K.J., Merchant M.J. (2017). Modelling direct DNA damage for gold nanoparticle enhanced proton therapy. Nanoscale.

[31] Chow J.C.L. (2011). A performance evaluation on Monte Carlo simulation for radiation dosimetry using cell processor. J. Comp. Meth. Sci. Eng..

[32] McMahon S.J., Hyland W.B., Muir M.F., Coulter J.A., Jain S., Butterworth K.T., Schettino G., Dickson G.R., Hounsell A.R., O’sullivan J.M. (2011). Biological consequences of nanoscale energy deposition near irradiated heavy atom nanoparticles. Sci. Rep..

[33] Sung W., Ye S.J., McNamara A.L., McMahon S.J., Hainfeld J., Shin J., Smilowitz H.M., Paganetti H., Schuemann J. (2017). Dependence of gold nanoparticle radiosensitization on cell geometry. Nanoscale.

[34] Rogers D.W., Kawrakow I., Seuntjens J.P., Walters B.R., Mainegra-Hing E. (2003). NRC User Codes for EGSnrc.

[35] Rogers D.W. (2006). Fifty years of Monte Carlo simulations for medical physics. Phys. Med. Biol..

[36] Kim J.H., Hill R., Kuncic Z. (2012). An evaluation of calculation parameters in the EGSnrc/BEAMnrc Monte Carlo codes and their effect on surface dose calculation. Phys. Med. Biol..

[37] Moradi F., Saraee K.R., Sani S.A., Bradley D.A. (2021). Metallic nanoparticle radiosensitization: The role of Monte Carlo simulations towards progress. Rad. Phys. Chem..

[38] Chow J.C.L., Owrangi A.M. (2012). Surface dose reduction from bone interface in kilovoltage x-ray radiation therapy: A Monte Carlo study of photon spectra. J. Appl. Clin. Med. Phys..

[39] Rogers D.W., Walters B., Kawrakow I. (2009). BEAMnrc Users Manual.

[40] Walters B.R., Kawrakow I., Rogers D.W. (2005). DOSXYZnrc Users Manual.

[41] Chow J.C.L., Ficai A., Grumezescu A.M. (2017). Dose Enhancement Effect in Radiotherapy: Adding Gold Nanoparticle to Tumour in Cancer Treatment. Nanostructures for Cancer Therapy.

[42] Cooper D.R., Bekah D., Nadeau J.L. (2014). Gold nanoparticles and their alternatives for radiation therapy enhancement. Front. Chem..

[43] Leung M.K., Chow J.C.L., Chithrani B.D., Lee M.J., Oms B., Jaffray D.A. (2011). Irradiation of gold nanoparticles by x-rays: Monte Carlo simulation of dose enhancements and the spatial properties of the secondary electrons production. Med. Phys..

[44] Chow J.C.L. (2020). Depth dose enhancement on flattening-filter-free photon beam: A Monte Carlo study in nanoparticle-enhanced radiotherapy. Appl. Sci..

